# Audit on Naloxone Dispensing for Clients Prescribed Opioid Substitution Treatment (OST) in the CGL Service in East Sussex

**DOI:** 10.1192/bjo.2025.10573

**Published:** 2025-06-20

**Authors:** Daniel Di Francesco

**Affiliations:** Sussex Partnership NHS Foundation Trust, Eastbourne, United Kingdom

## Abstract

**Aims:** To identify any patients on OST who have not been offered or received naloxone.

To improve documentation of naloxone provision.

To explore reasons why clients have declined naloxone.

The standard audited against was that 100% of clients prescribed OST should be offered naloxone. This is advised in the Department of Health publication “Drug misuse and dependence, UK guidelines on clinical management”, which advises services should be “offering all opiate users in the community access to a take-home supply of naloxone with instructions on its use”.

CGL is a charity which provides medical and psychosocial support for people who are affected by alcohol and drugs. As of January 2025, they prescribe Opiate Substitute Treatment (OST), typically a formulation of methadone or buprenorphine, to 748 clients in East Sussex. Naloxone is an opioid antagonist which can reverse the effects of opiate overdose, and is offered to service users to reduce mortality from overdose.

**Methods:** It was recorded for each of the 748 clients whether they had been offered naloxone and training, and whether they had accepted. The data were collected from the CGL County-wide Opiate report in January 2025, and cross-referenced with a manual review of notes on Criis, the electronic clinical notes platform used by CGL.

**Results:** Of the 748 clients prescribed OST, 65 total clients did not have naloxone (8.7%), while 60 had expired naloxone. 54 clients did not have naloxone documented in the opiate report, but on a manual review of notes it was confirmed to have been given, but the dispensing form had not been completed on Criis. Of the 65 clients without naloxone, 100% had been offered naloxone and had declined.

**Conclusion:** The manual review of notes showed that naloxone uptake was better than the opiate report suggested, due to a lack of coding. Most commonly, this was a result of clients already having naloxone from another service or from earlier course of treatment. A potential barrier is the data entry required – if a client was dispensed from another location, it must also be confirmed by the keyworker and manually entered into a form on Criis.

The keyworkers of those clients with expired naloxone were informed, to arrange suitable follow-up.

The most common reason for declining naloxone was that the client was no longer injecting, and had no contacts who used opiates (57%). Also mentioned was the stigma of carrying naloxone, and the fear that others would assume they had relapsed.

